# Experimental Study on the Mechanical Performance of Large Plate Girders Under Eccentric Loading

**DOI:** 10.3390/ma19194223

**Published:** 2026-10-05

**Authors:** Yuxiang Zhang, Xiufeng Chen, Jinli Wei, Ju Chen

**Affiliations:** 1 School of Intelligent Transportation and Vehicle, Qingdao University of Technology, 777 Jialingjiang Road, Huangdao District, Qingdao 266520, China; zhangyuxiang@qut.edu.cn (Y.Z.); chenxiufeng@qut.edu.cn (X.C.); jinliwei@qut.edu.cn (J.W.); 2 College of Civil Engineering and Architecture, Zhejiang University, 866 Yuhangtang Road, Xihu District, Hangzhou 310058, China

**Keywords:** large plate girder, high-strength bolted connection, short deep beam, boiler steel structure

## Abstract

To investigate the mechanical performance of large plate girders used in boiler-supporting steel structures under eccentric loading, nine 1:5-scaled closed-section HK-type and open-section π-type specimens were designed and tested. The specimens had a span of 5960 mm, an overall length of 6220 mm, and a depth of 1760 mm. Concentric loading and eccentric loading with eccentricities of 30 mm, 60 mm, and 90 mm were considered. The results show that under eccentric loading, the mid-span displacement increased with increasing eccentricity for both HK-type and π-type specimens. Compared with π-type specimens, HK-type specimens exhibited smaller mid-span displacements and higher secant stiffness under the same eccentric loading conditions, indicating better deformation resistance. At a load level of 3000 kN, the secant stiffness of the HK-type specimen decreased from 192.31 kN/mm to 172.81 kN/mm as the eccentricity increased from 0 mm to 90 mm, while that of the π-type specimen decreased from 156.57 kN/mm to 142.18 kN/mm. The measured transverse brace stresses of π-type specimens were higher than those of HK-type specimens, indicating stronger interaction between the π-type girder and the lateral-restraint system under the applied eccentric-loading conditions. Under the adopted eccentric loading and lateral restraint conditions, the HK-type specimens exhibited smaller mid-span displacements, higher absolute secant stiffness, and lower transverse-brace stresses than the corresponding π-type specimens. This study provides experimental data for understanding the deformation behavior, torsional response, and stiffness degradation of large plate girders in boiler-supporting steel structures.

## 1. Introduction

With the continuous development of large-capacity and ultra-supercritical thermal power generating units, the scale of boiler-supporting steel structures has increased significantly in terms of height, span, and load level [1,2,3]. Large plate girders, as the primary horizontal load-bearing members at the top of the boiler-supporting steel structure, are responsible for supporting the suspended loads of the boiler body, heating surfaces, and auxiliary equipment. Their mechanical performance directly affects the safety, reliability, and serviceability of the entire boiler-supporting steel structure [1,4].

To meet the increasing demands for load-carrying capacity, modern large plate girders have gradually evolved toward longer spans, deeper sections, heavier loads, and more complex connection details [3,5]. For 1000 MW ultra-supercritical units, the span of large plate girders typically exceeds 30 m, with section depths up to 10 m, resulting in small span-to-depth ratios. Such characteristics make these members behave as typical short deep beams rather than conventional slender beams [6,7], where shear deformation, local stress redistribution, and web participation play critical roles in the overall load-transfer mechanism [8,9].

Due to fabrication, transportation, and site erection constraints, large plate girders are often manufactured in segments and assembled on site [5,10]. Segmental fabrication introduces complex mechanical phenomena, including shear transfer between webs, slip at composite interfaces, and force redistribution within bolted connection groups [11]. To ensure structural integrity, connections are generally realized using splice plates, welding, and high-strength friction bolts, whose mechanical performance directly affects global stiffness, force-transfer efficiency, and structural safety [12].

Previous studies have reviewed boiler-supporting steel structure technology and the performance of large plate girders. Liu et al. [1] summarized the development and future trends of boiler steel structures. Zhao [5] compared Chinese and American design methods for segmented large plate girders, highlighting differences in segment arrangement, connection detailing, and strength verification. Hu et al. [7] investigated uneven foundation settlement effects on boiler steel structures, demonstrating internal force redistribution and local stress changes. Zhang and Zhao [4] studied cantilevered spliced girders through scaled experiments and finite element analysis, revealing interactions between web buckling and splice bolt behavior. Xie [6] analyzed fabrication issues of large plate girders, including camber control, splicing hole alignment, and deformation management. These studies provide essential references for understanding large plate girder performance.

From a structural mechanics perspective, large plate girders are thin-walled members composed of flange plates, webs, and stiffeners, with behavior governed by bending, shear, local web buckling, and post-buckling resistance [10]. Tetougueni et al. [10] reviewed steel plate girder behavior under in-plane loading, emphasizing the effects of web buckling and stiffener configuration. Luo et al. [13] studied high-strength steel plate girders under patch loading and proposed design recommendations. Chacón et al. [12] indicated that initial imperfections and boundary conditions significantly influence numerical simulations. Maiorana et al. [14] analyzed the interaction of bending moment, shear, and local loading, highlighting the nonlinearity in deep plate girder behavior.

Large plate girders have been increasingly adopted in large boiler-supporting steel structures to improve flexural stiffness and load-carrying capacity while satisfying segmented fabrication and transportation requirements [15]. HK-type large plate girders, consisting of upper and lower girder segments, dual webs, stiffeners, and high-strength bolts, exhibit complex three-dimensional stress distributions, where bending, web interaction, load transfer across connections, and local stability interact simultaneously. Asymmetric loading caused by installation deviations or operational variations may generate torsional effects and uneven stress distributions across webs, influencing stiffness and stability [15].

High-strength friction bolts are widely used for segmental connections of large plate girders, and their mechanical performance is critical for collaborative behavior. Previous studies highlighted differences between Chinese and American design standards for segmental design and bolted connection calculations. The American Specification for Structural Steel Buildings (ANSI/AISC 360-16 [16]) predicts larger individual bolt capacities, resulting in fewer required bolts, whereas the Chinese Standard for Design of Steel Structures (GB 50017-2017 [17]) adopts a more conservative approach emphasizing friction-type load transfer and higher safety factors. Shi [11] compared bolted connection provisions in Chinese and American standards and revealed differences in resistance evaluation. Maiorana and Pellegrino [18] further showed that different international design philosophies lead to notable discrepancies in predicted connection capacities. Teh and Clements [19] investigated shear-out failure mechanisms of bolted connections and proposed methods for estimating ultimate resistance.

Although previous studies have addressed the general behavior of steel plate girders, bolted splice connections, and boiler-supporting steel structures, experimental information remains limited for full-depth segmented boiler girders subjected to eccentric vertical loading. In particular, comparative measurements of the vertical deformation, asymmetric web response, and transverse-restraint demand of closed HK-type and open π-type configurations under the same loading and restraint conditions are scarce. Therefore, the principal research question of this study is: how do the measured deformation, web-strain response, transverse-brace stress, and secant stiffness of HK-type and π-type segmented large plate girders differ as the loading eccentricity increases under a prescribed lateral-restraint system? The experimental program was designed to provide comparative evidence for selecting and evaluating these two practical girder configurations when eccentric loading and hanger-rod opening requirements must be considered.

**Figure 1:**
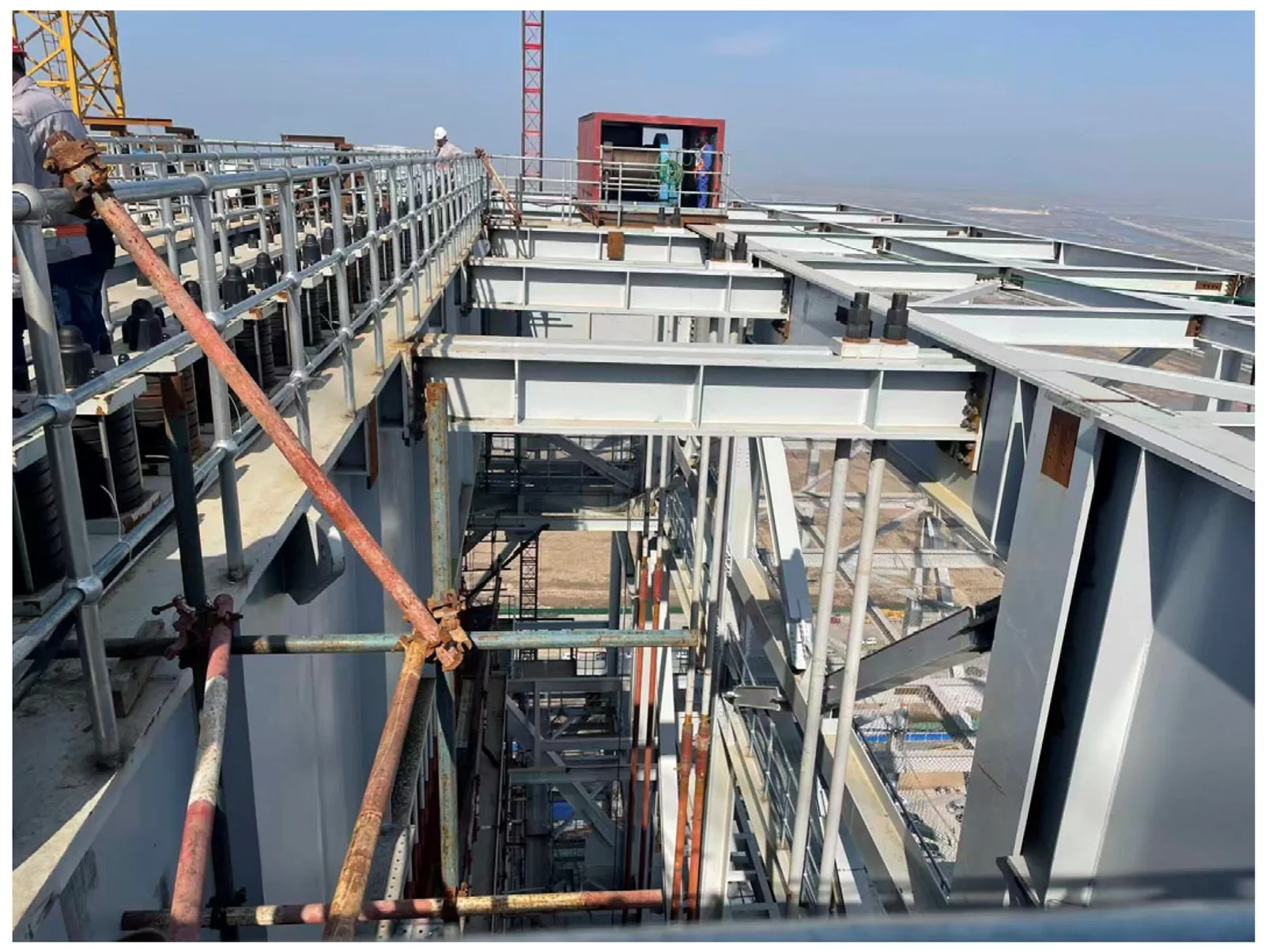
Typical application of a large plate girder in a boiler-supporting steel structure.

Large plate girders used in boiler-supporting structures may adopt different cross-sectional configurations depending on load-transfer requirements, fabrication constraints, and the need to accommodate suspended components. Among the commonly used configurations, closed or box-like girders provide a relatively enclosed load-carrying path and generally exhibit high sectional rigidity, whereas open or partially open girders provide greater accessibility for hanger rods, piping, and erection operations but may exhibit a different deformation and restraint response under eccentric loading. In the present study, two practical configurations are considered: the HK-type girder, which has a closed sectional form, and the π-type girder, which has an open sectional form with a central opening to accommodate hanger-rod and construction requirements. These two configurations therefore represent different practical compromises between structural rigidity and functional accessibility. Figure 1 shows a typical application of a large plate girder in a boiler-supporting steel structure.

Due to the enormous size of large plate girders, full-scale experiments are expensive and challenging. Therefore, scaled experimental testing was adopted in this study to investigate the response of HK-type and π-type large plate girders under concentric and eccentric loading. The experimental program focused on the measured displacement response, web strain distribution, transverse-brace response, and secant stiffness under the adopted loading and restraint conditions. The results provide experimental observations for understanding the behavior of these two girder configurations and serve as a basis for further analytical and numerical investigations.

The novelty of this study lies in the direct experimental comparison of two practical segmented large plate-girder configurations, namely the closed HK-type and open π-type girders, under identical concentric and eccentric loading and lateral-restraint conditions. Unlike previous studies focusing mainly on conventional plate-girder behavior, connection design, or numerical analysis, the present tests provide comparative measurements of mid-span deformation, asymmetric web-strain response, transverse-brace stress, and secant stiffness for these two boiler-girder configurations as the loading eccentricity increases. The significance of the study is that these measurements provide experimental evidence for understanding how the two practical configurations respond differently to eccentric loading and interact with the lateral-restraint system. The results can therefore serve as a reference for configuration selection and for subsequent analytical and numerical investigations of large segmented boiler girders, within the limitations of the tested scale, loading range, and restraint conditions.

## 2. Experimental Investigation

### 2.1. Design of Specimens

Due to the limitations of the testing equipment and laboratory space, as well as considerations related to the arrangement and fabrication of bolts at the composite interface, the scaled large plate girder was designed with a span of 5960 mm, a total length of 6220 mm, and a height of 1760 mm.

**Figure 2:**
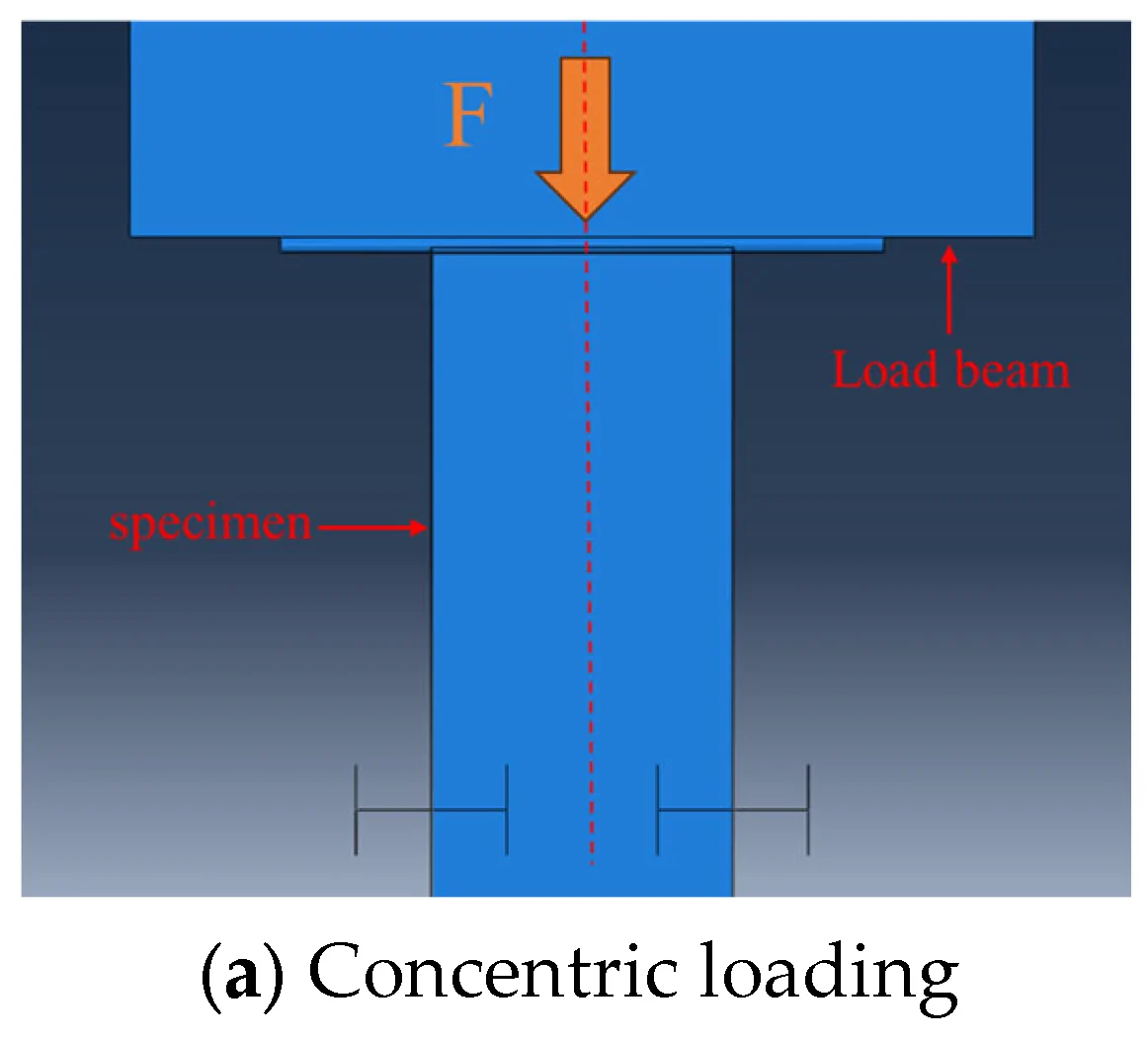

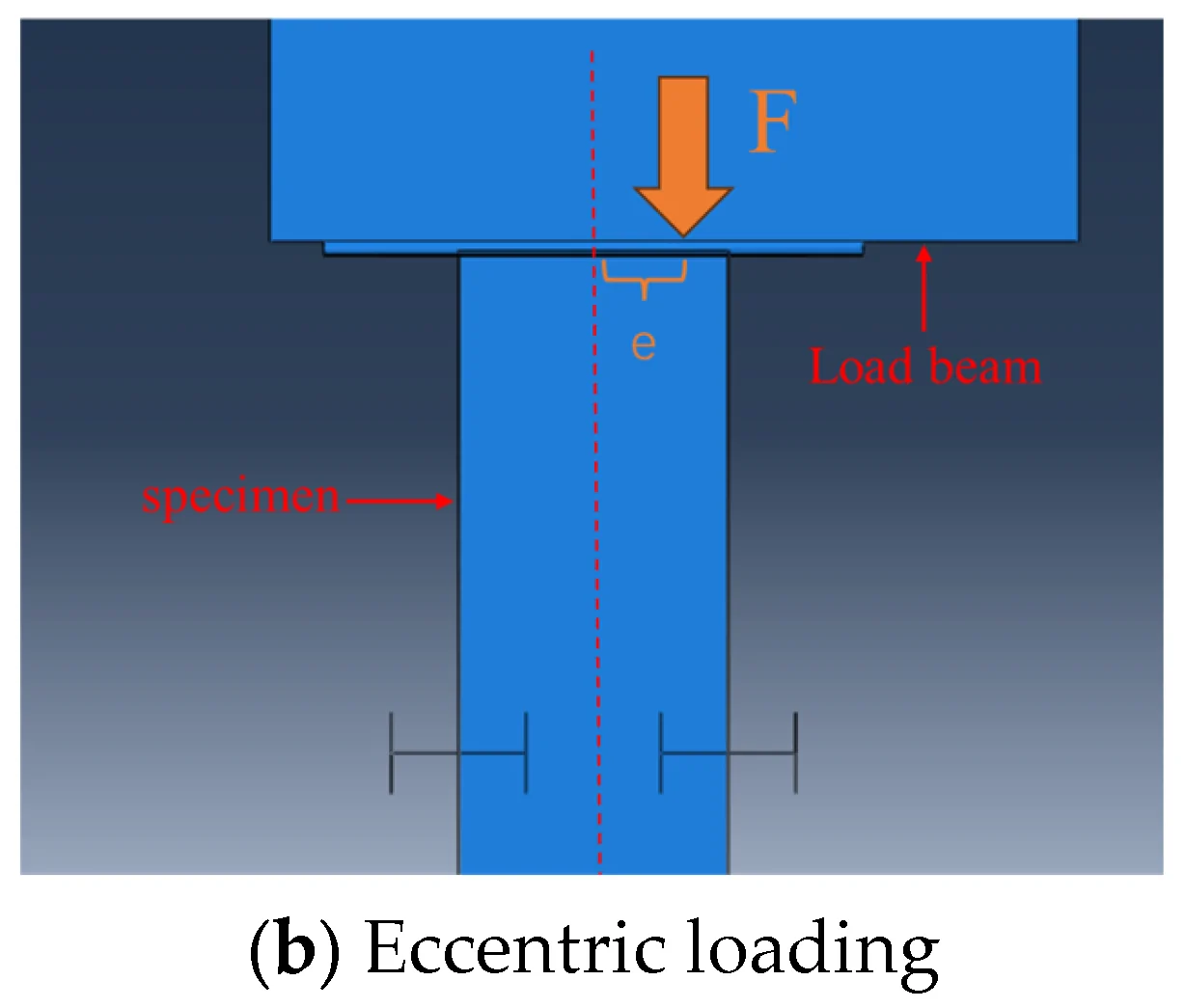
Loading scheme.

In the specimen designation “HK-*e*-*F*”, *e* represents the transverse distance between the vertical load line and the section centerline at the loading location, while *F* represents the final applied load value, as illustrated in Figure 2. For example, “HK-0-3000” denotes an HK-type large plate specimen subjected to concentric loading with a loading value of 3000 kN. This naming convention enables clear identification of specimen types and loading parameters under different loading conditions, facilitating subsequent experimental data processing and analysis. Detailed specimen information is summarized in Table 1.

**Figure 3:**
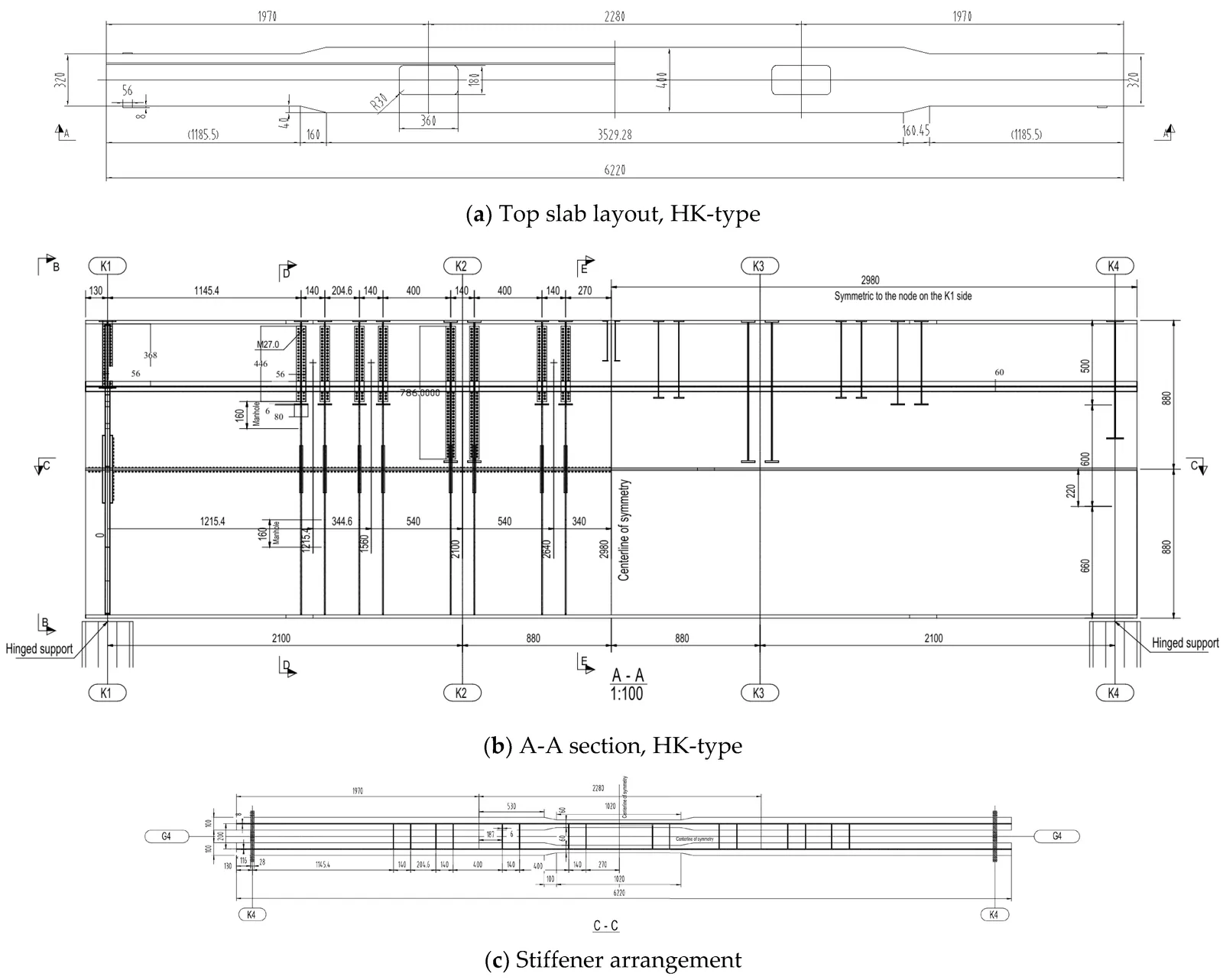

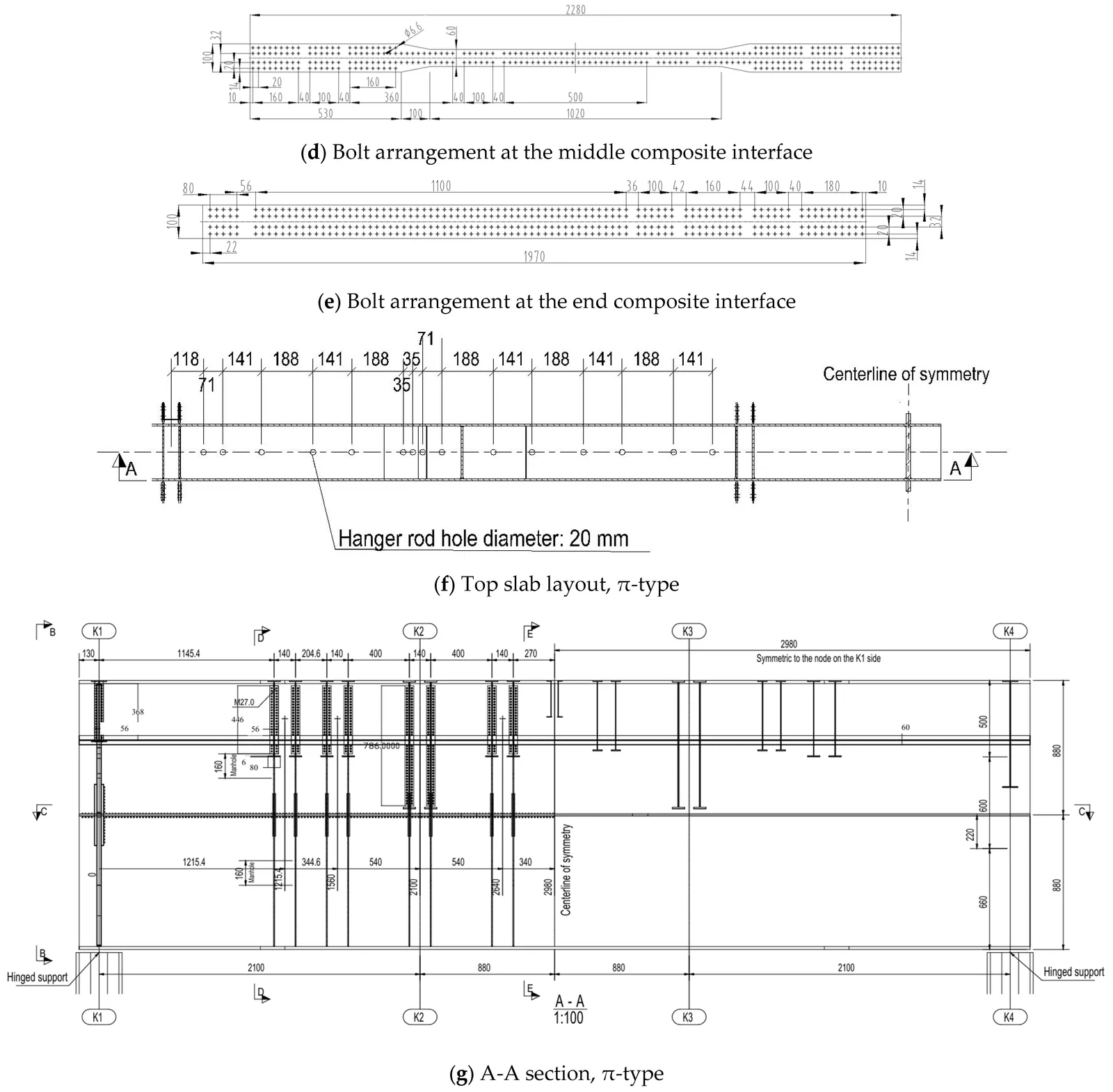
Layout drawing of the specimen (mm).

The drawings of the HK-type specimen are shown in Figure 3a–e, while those of the π-type specimen are shown in Figure 3f,g. Equal-strength design principles were adopted for bolt selection. In the original model, two bolt specifications, namely M27 and M30 bolts of grade 10.9 (Ningbo Dongnan High-Strength Fastener Co., Ltd., Ningbo, Zhejiang, China), were used. According to Table 5 in Chapter 7 of GB/T 3098.1–2010 Mechanical Properties of Fasteners—Bolts, Screws and Studs [20], the relevant parameters are as follows: the M27 bolt has an effective cross-sectional area of 459 mm^2^ and a proof load of 381 kN, while the M30 bolt has an effective cross-sectional area of 561 mm^2^ and a proof load of 466 kN.

To further clarify the basis of comparison between the two tested structural systems, the basic geometric sectional properties were calculated from the specimen fabrication drawings and are summarized in Table 2. For the π-type configuration, the representative section was obtained from the HK-type section by removing the central portion of the 20-mm-thick bottom plate between the inner faces of the two webs. The discrete hanger-rod holes in the top plate were not included in the representative cross-section calculation because they constitute localized openings. The results show that the π-type section has approximately 91.5% of the gross steel area and 83.2% of the major-axis second moment of area of the HK-type section.

Since the geometric dimensions of the model were scaled down to one-fifth of the prototype, the corresponding load scale ratio was 1/25 of the full-scale structure. Therefore, when the number of bolts remains the same as that in the original model and the bolt grade is not reduced, the effective cross-sectional area of the selected bolts should not be less than 1/25 of the effective cross-sectional area of the corresponding bolts in the original model in order to satisfy the shear stress requirement. Similarly, to satisfy the tensile force requirement, the minimum proof load of each bolt should also not be less than 1/25 of the corresponding proof load in the original model.

A geometric scale factor of 1:5 was adopted for the test specimens. Accordingly, the model span, overall length, and depth of 5960, 6220, and 1760 mm correspond to nominal prototype dimensions of 29.80, 31.10, and 8.80 m, respectively. The loading eccentricities of 0, 30, 60, and 90 mm correspond to prototype eccentricities of 0, 150, 300, and 450 mm. Because the prototype and scaled specimens were fabricated using steel with comparable elastic properties, the elastic modulus, stress, and strain scale factors were taken as unity. Based on dimensional similitude, the length, area, force, displacement, and secant-stiffness scale factors were 1/5, 1/25, 1/25, 1/5, and 1/5, respectively. The corresponding bending-moment and torque scale factors were both 1/125, while the second moment of area and torsional constant scale factors were 1/625. Therefore, the adopted 1/25 load scale is consistent with the requirement of maintaining comparable nominal stress levels in the elastic bending, shear, and torsional responses of geometrically similar steel members. Under this similitude relation, model loads of 2500 and 3000 kN correspond nominally to prototype-equivalent loads of 62.5 and 75 MN, respectively. These values are reported only to indicate the force–scale relationship and should not be interpreted as specific service or design loads of a prototype boiler girder. The experimental load levels were selected according to the objectives and loading-control requirements of the present comparative test program, as described in Section 2.3.

For local plate behavior, geometric similitude requires the plate slenderness ratios and the relative stiffener spacing to remain unchanged. Therefore, the principal web, flange, and stiffener dimensions of the scaled specimens were selected to reproduce the corresponding geometric proportions of the prototype as closely as permitted by commercially available plate thicknesses and fabrication requirements. Any unavoidable deviations in plate thickness, local slenderness, and stiffener spacing are regarded as scale distortions that may influence local buckling behavior. In addition, initial geometric imperfections, welding-induced residual stresses, local contact deformation, and surface roughness cannot be scaled strictly according to the geometric scale. Their possible influence is therefore recognized as a limitation of the present scaled experimental investigation.

For the M27 bolt, 1/25 of the effective cross-sectional area is 18.36 mm^2^, and 1/25 of the proof load is 15.24 kN. For the M30 bolt, 1/25 of the effective cross-sectional area is 22.44 mm^2^, and 1/25 of the proof load is 18.64 kN. According to the specifications of M7 bolts, a grade 10.9 M7 bolt has an effective cross-sectional area of 28.9 mm^2^ and a proof load of 24 kN, both of which satisfy the design requirements. Therefore, grade 10.9 M7 bolts were adopted in the 1:5-scaled model to replace the M27 and M30 bolts in the corresponding locations of the original model.

The HK-type and π-type specimens were intended to represent two practical large plate-girder configurations rather than strictly equivalent comparison sections. Although the two configurations had comparable overall geometry and were tested under the same loading and restraint conditions, they were not specifically designed to have identical steel area, mass, bending strength, flexural stiffness, torsional stiffness, or warping resistance. Therefore, the smaller displacement measured for the HK-type specimens should not be attributed exclusively to the closed-section configuration. The observed differences should instead be interpreted as the overall response of the two tested structural systems, reflecting the combined effects of section geometry, flexural and torsional properties, local load-transfer mechanisms, connection behavior, and the adopted restraint conditions.

The plate thicknesses, stiffener arrangement, splice geometry, and bolt layout were defined according to the specimen fabrication drawings. However, some specimen-specific parameters, including the actual bolt pretension, interface slip factor, fabrication-induced geometric deviations, and initial geometric imperfections, were not independently measured during specimen fabrication. Therefore, their quantitative effects cannot be evaluated retrospectively, and no assumed values have been introduced in the revised manuscript. The possible influence of these parameters on local connection behavior and the measured structural response is acknowledged as a limitation of the present experimental study.

For each combination of girder configuration and loading eccentricity, one full girder specimen was tested; therefore, replicate tests were not conducted for the structural specimens. Replication was limited to the material tensile tests, for which three coupons were tested for each plate thickness. Consequently, the comparisons between the HK-type and π-type specimens are descriptive comparisons of the measured responses of individual specimens rather than statistical inferences regarding specimen populations.

### 2.2. Material Properties

**Figure 4:**
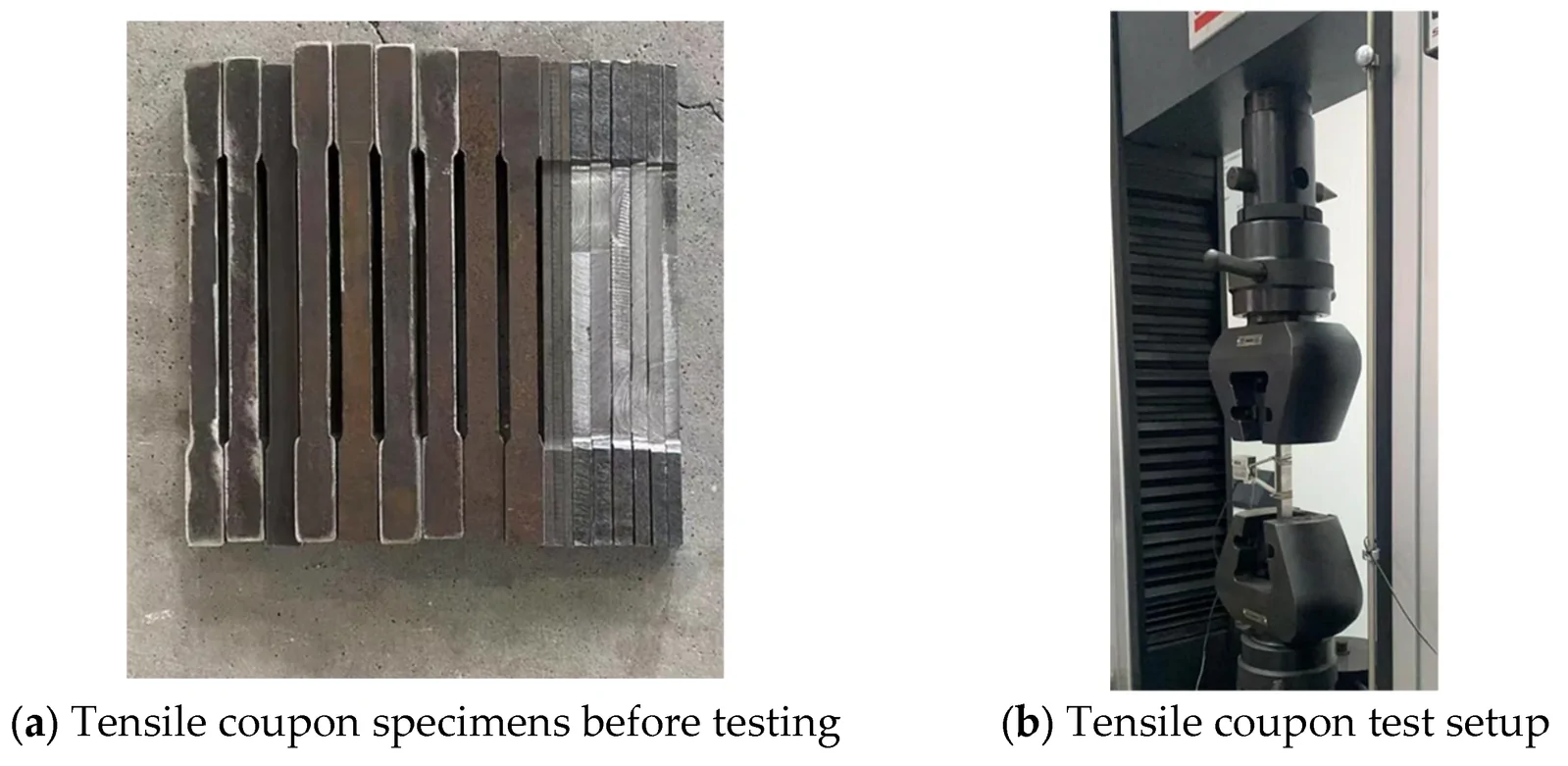
Tensile coupon tests.

The material property tests were conducted using a 300 kN universal testing machine Yi Test Equipment (Hangzhou) Co., Ltd., Hangzhou, Zhejiang, China) in the Materials Laboratory of Zhejiang University, and data acquisition was performed through the dedicated system integrated with the testing machine. The tests were carried out in accordance with the relevant provisions of Steel and Steel Products—Location and Preparation of Test Pieces for Mechanical Testing (GB/T 2975–1998 [21]) and Metallic Materials—Tensile Testing—Part 1: Method of Test at Room Temperature (GB/T 228.1–2010 [22]). The elastic modulus, yield strength, and yield strain of the material were measured using an extensometer, while the ultimate tensile strength and tensile strain were measured using a dial gauge. Photographs of the material property tests are shown in Figure 4. During the initial stage of the material property tests, no obvious sectional deformation was observed within the extensometer gauge length, and the specimens exhibited linear deformation with increasing load. As the load continued to increase, the specimens gradually yielded, and rapid sectional deformation occurred within the extensometer range until a sudden reduction in the cross-sectional area appeared at a certain location, accompanied by the occurrence of necking, which marked the end of the test.

**Figure 5:**
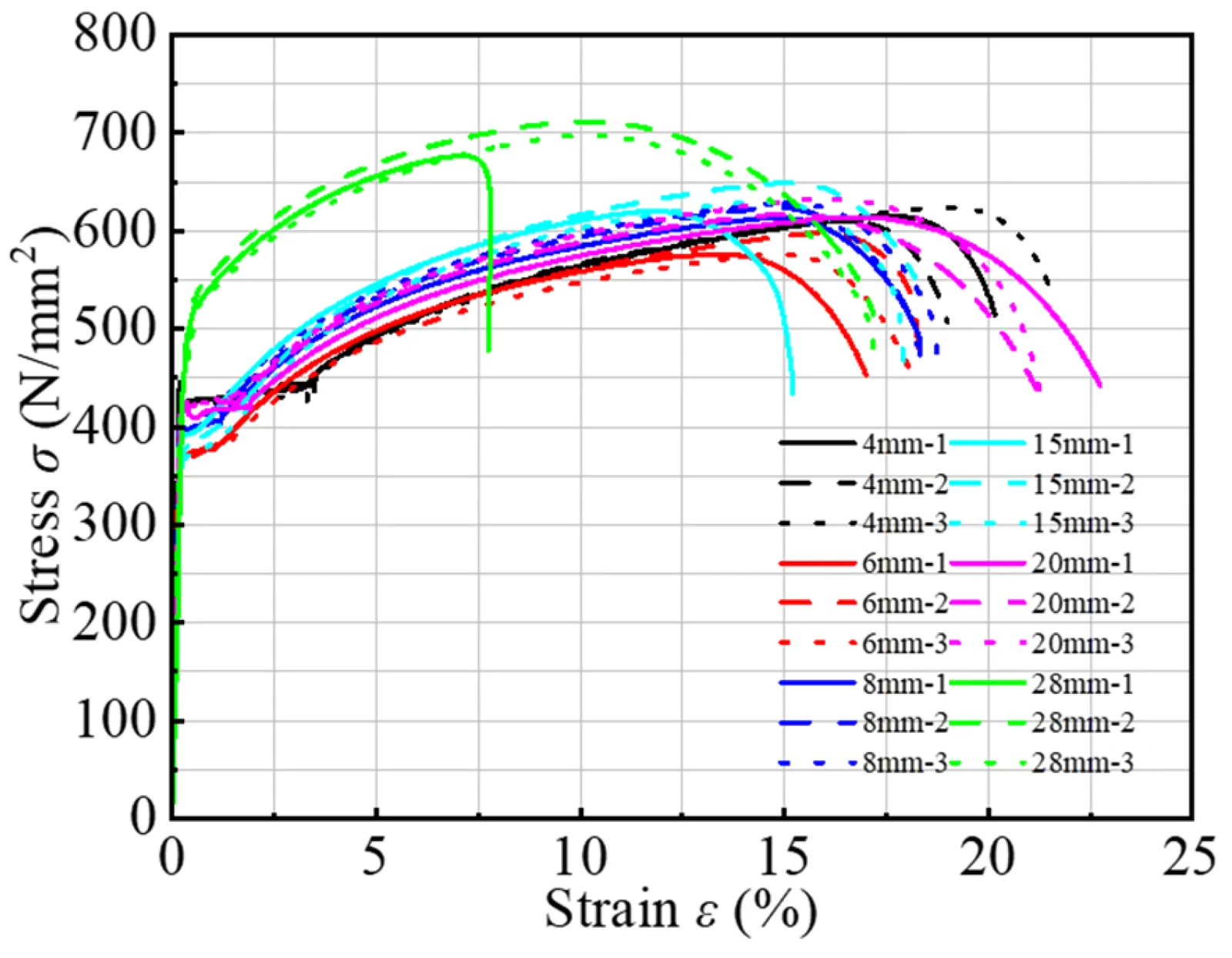
Stress–strain curves from tensile coupon tests.

After the tensile tests, the collected data were processed and summarized, and the material properties of the six types of steel plates are presented in Table 3. The material property data for each type of steel plate represent the average values obtained from three tensile specimens. The stress–strain curves are shown in Figure 5.

For each plate thickness, three tensile coupon specimens were tested. Table 3 presents the individual values of elastic modulus *E*, yield strength *f_y_*, and ultimate tensile strength *f_u_*, together with their arithmetic mean, sample standard deviation (SD), and coefficient of variation (CV). The CV values of *E*, *f_y_*, and *f_u_* were within 0.63–0.98%, 2.38–3.41%, and 1.92–3.45%, respectively, indicating relatively limited dispersion among the three coupons for each plate thickness. Given the small sample size (*n* = 3), these statistics are used only as descriptive measures of repeatability rather than for inferential statistical analysis.

### 2.3. Test Setup and Procedure

**Figure 6:**
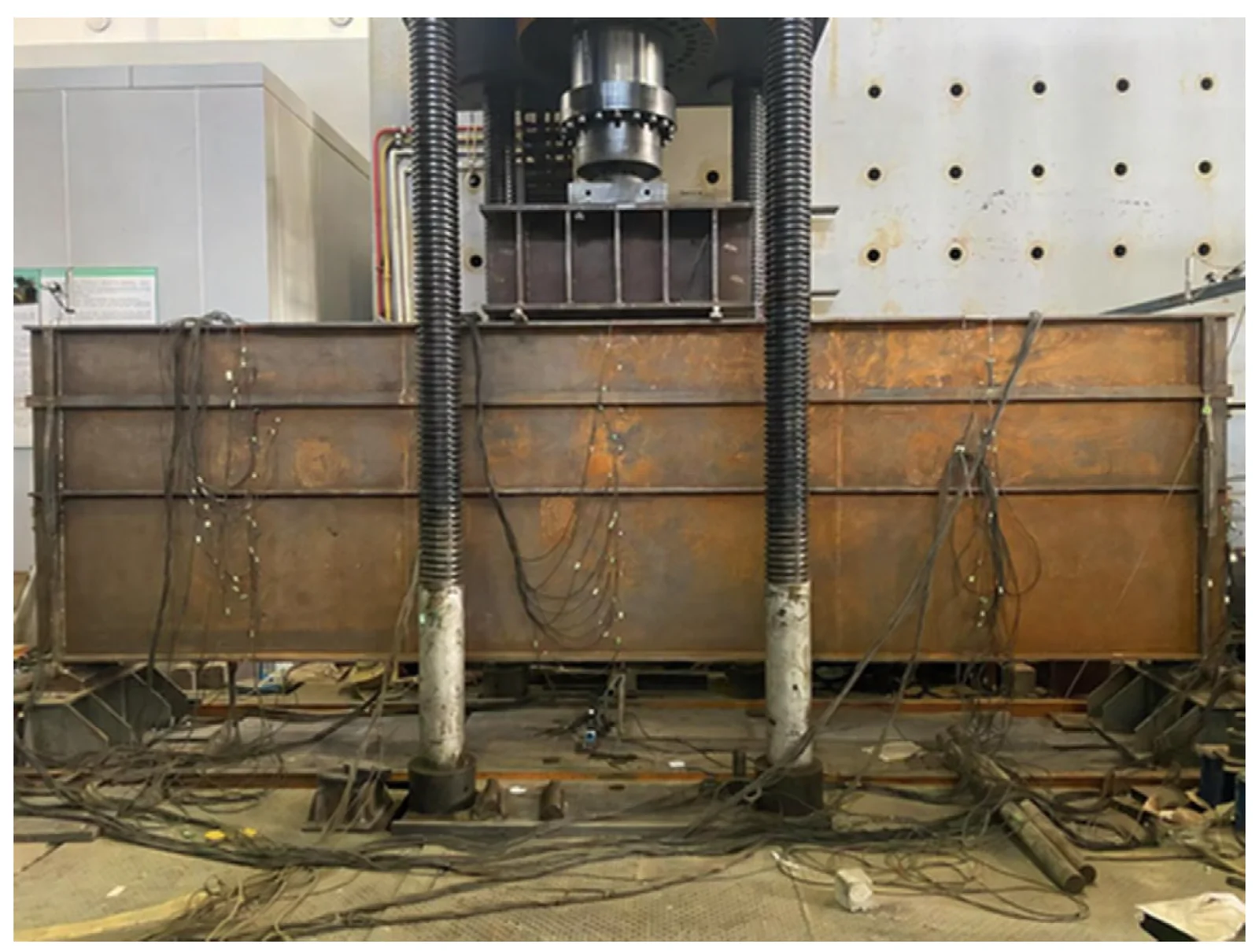
Test setup.

The tests were conducted using a YAW-10000F computer-controlled electro-hydraulic servo multifunctional testing machine (Jinan Shijin Group Co., Ltd., Jinan, Shandong, China) at Zhejiang University. The specimens were tested as simply supported girders under two-point vertical loading. The load generated by the hydraulic actuator was transferred through a distribution beam to two loading points spaced 1.0 m apart. Under eccentric loading, the loading system was laterally offset by the prescribed eccentricity *e*, so that the vertical resultant did not coincide with the section centerline. The load was therefore transferred from the actuator through the distribution beam and loading contact regions to the girder and subsequently to the two end supports, as shown in Figure 6.

**Figure 7:**
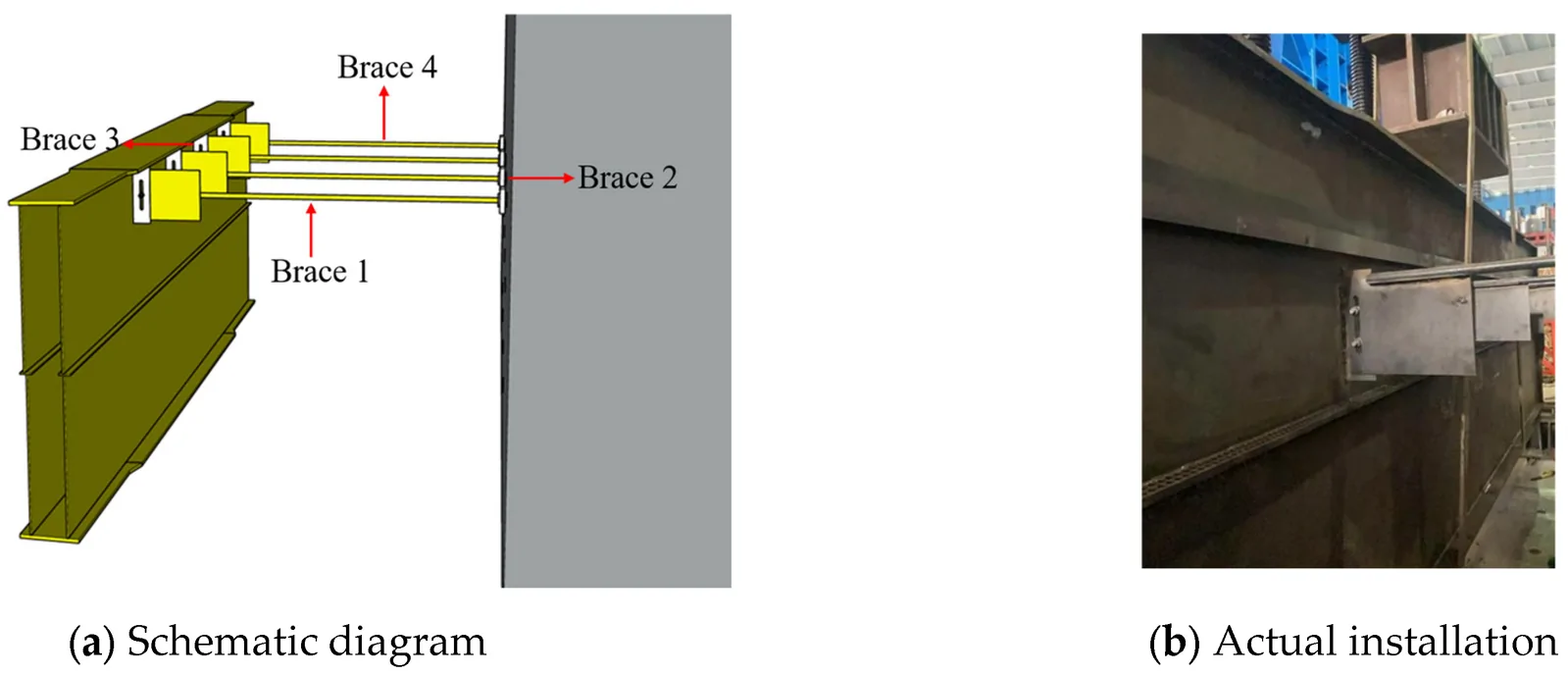
Transverse bracing system.

To prevent excessive out-of-plane displacement and lateral overturning during eccentric loading, four transverse braces were installed and connected to the reaction wall, as shown in Figure 7. Strain gauges were attached to the braces to monitor their axial strain response, from which the corresponding brace stresses were obtained. Because measurable stresses developed in the braces during testing, the braces participated in the lateral force-transfer mechanism and provided non-negligible restraint to the specimens. Therefore, the measured response should be interpreted as the behavior of the girder–restraint system under the adopted eccentric loading conditions rather than as the free torsional response of an unrestrained girder. Accordingly, the brace-stress measurements were used to characterize the interaction between the girder and the lateral restraint system.

**Figure 8:**
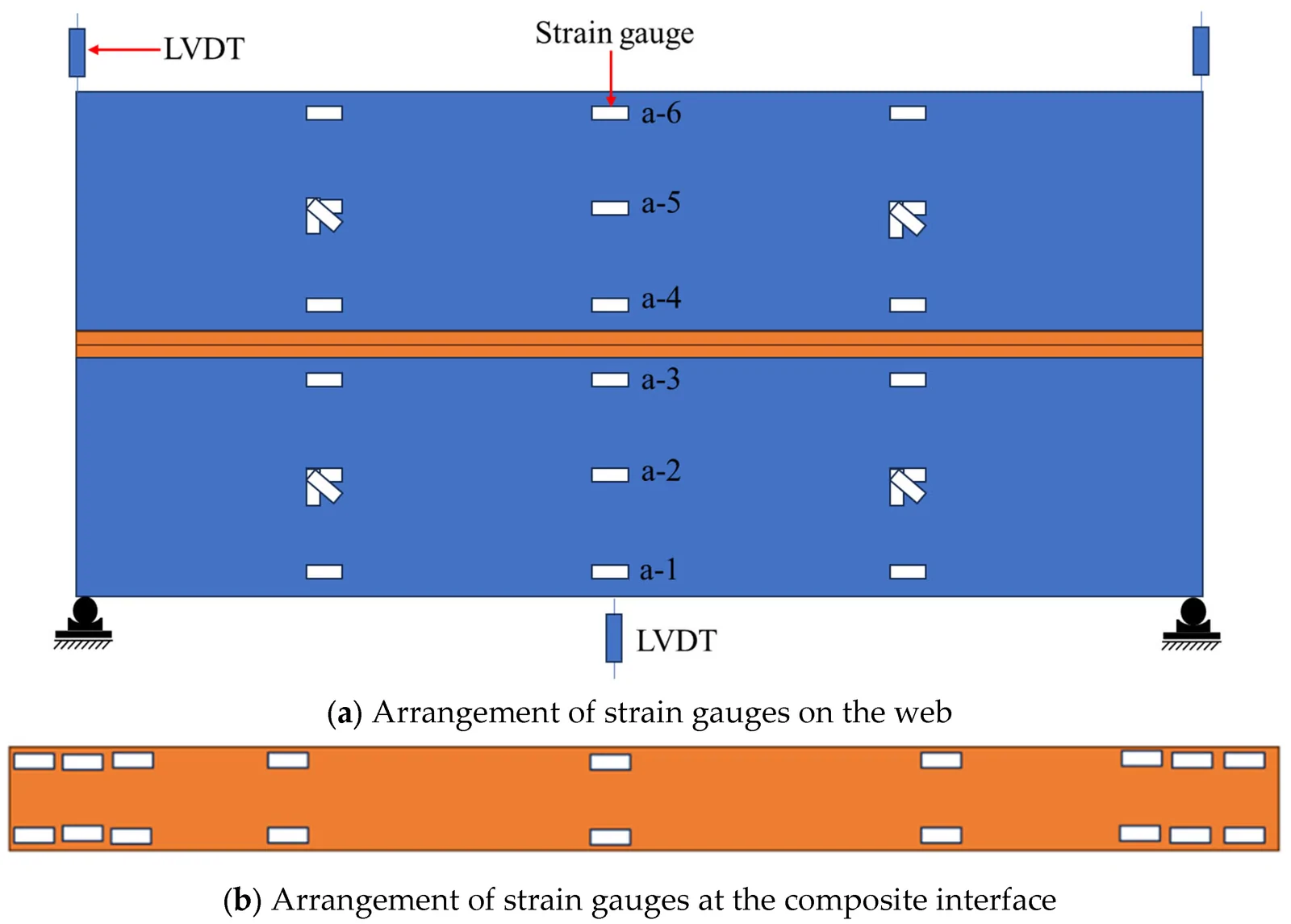
Measurement point arrangement.

As shown in Figure 8, three LVDTs (Linear Variable Differential Transformers) were installed across the bottom flange at the mid-span section, with one located at the transverse centerline and the other two located near the two outer edges of the bottom flange. The centerline LVDT was used to obtain the mid-span vertical displacement. Under eccentric loading, the edge LVDT located on the side toward which the loading line was offset is referred to as the “near-side” displacement measurement, whereas the LVDT located at the opposite edge is referred to as the “far-side” displacement measurement. Thus, the terms “displacement near the concentrated load” and “displacement away from the concentrated load” used in Table 4 correspond to the vertical displacements measured by these two edge LVDTs, respectively. The displacement measurement points at the supports were arranged at the top of the specimen and were used to monitor support settlement for correction of the measured girder deformation.

Figure 8a,b indicate the locations of strain gauges on the web and at the composite interface, respectively. Through these precise displacement and strain measurements, the deformation response of the specimen under different loading conditions can be accurately evaluated.

A preload of 100 kN was applied. The specimen was loaded to the target value at a rate of 0.5 mm/min and held for 3 min, followed by unloading to zero load at the same rate. This procedure was used to eliminate clearances in the test setup, verify the coordinated performance of the testing equipment, and confirm through strain gauge monitoring that the specimen remained in the elastic working state.

During formal loading, the hydraulic actuator was operated under displacement control. Before the applied load reached 2500 kN, the actuator displacement rate was maintained at 1.0 mm/min. The loading process was divided into nominal 500 kN intervals, and the load was held for 5 min at each prescribed level for data acquisition and observation of the structural response. During this stage, the flange and web strains were monitored to confirm that the specimens remained predominantly within the elastic range.

After the applied load exceeded 2500 kN, displacement control was maintained, while the actuator rate was reduced to 0.5 mm/min. As the prescribed comparison load of 3000 kN was approached, the loading intervals were refined to approximately 50 kN and the holding time was extended to 10 min. The web-strain development and the load–displacement response were monitored continuously during this stage.

For the eight comparative specimens, loading was terminated after the prescribed final load of 3000 kN was reached, provided that no abnormal structural response occurred beforehand. Specimen HK-0-3800 was intentionally loaded beyond 3000 kN to investigate the transition toward elastic–plastic behavior. During the extended loading stage, the test was terminated when a clear abrupt change in the local web-strain response or another abnormal structural response was observed.

### 2.4. Test Results

**Figure 9:**
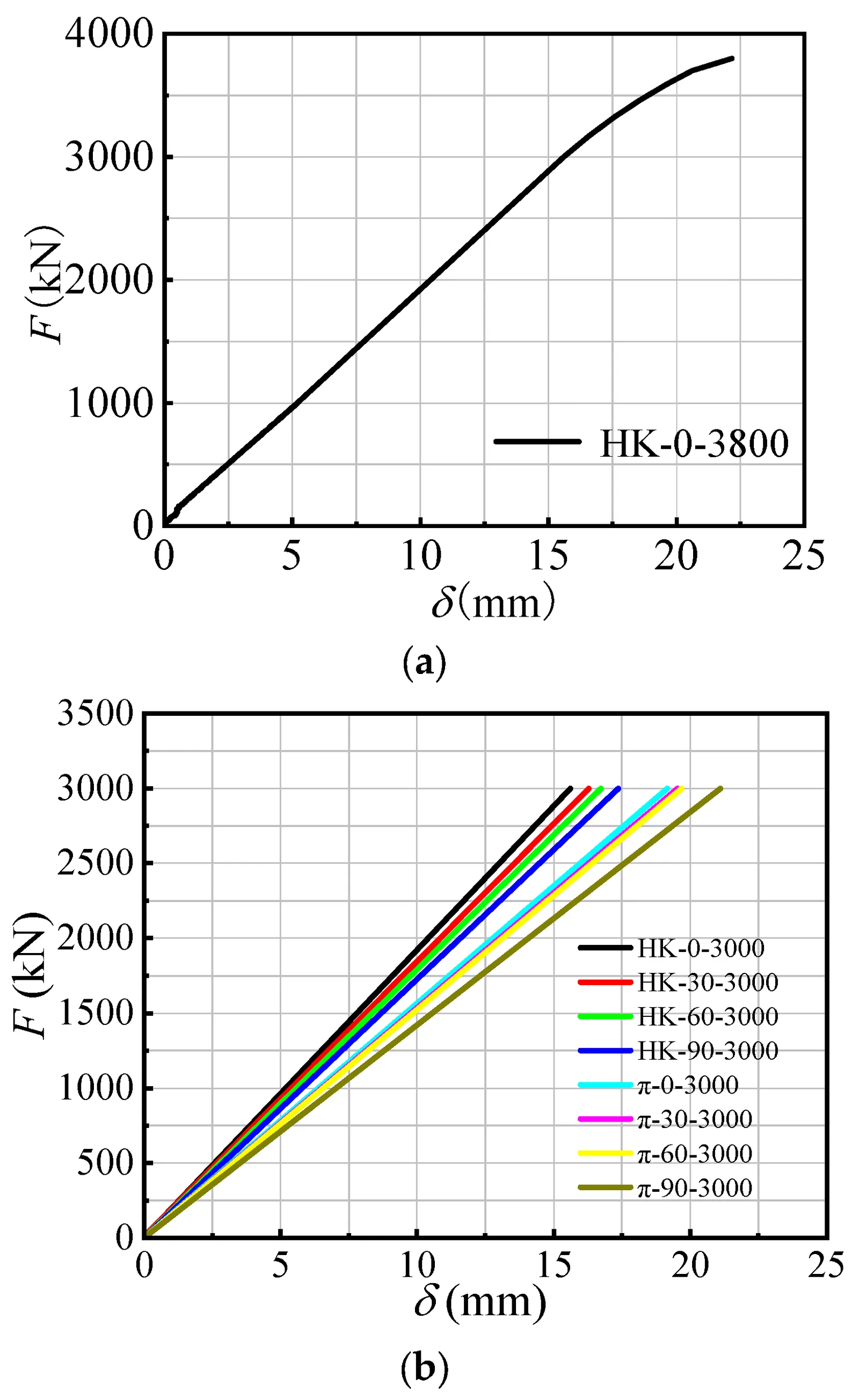
Load–displacement curves. (**a**) HK-0-3800 specimen; (**b**) HK-type and π-type specimens under different loading eccentricities.

For the tests intended to characterize the elastic response under different eccentricities, 3000 kN was selected as the prescribed final load. The purpose of these tests was to compare the deformation, strain, and brace response of the two configurations under a common load level rather than to determine their ultimate capacities. The load–displacement curves of the specimens under different loading conditions are shown in Figure 9.

Eccentric loading causes different mid-span displacements on the two sides of the beam and induces both bending and torsion. As a result, the beam undergoes not only flexural deformation but also possible twisting deformation at the mid-span, leading to a larger mid-span displacement compared with that under concentric loading. As shown in Table 4, the maximum mid-span displacement gradually increases with increasing eccentricity.

As shown in Table 4, the mid-span displacement of both HK-type and π-type specimens generally increased with increasing loading eccentricity. Under the same loading and restraint conditions, the HK-type specimens consistently exhibited smaller mid-span displacements than the corresponding π-type specimens. This observation is consistent with the generally higher torsional rigidity expected for a closed section. However, because the two configurations were not strictly identical in all sectional and connection properties, the measured displacement differences cannot be attributed exclusively to torsional stiffness or section openness. Instead, the observed response should be interpreted as the combined effect of section geometry, flexural and torsional properties, local load-transfer mechanisms, connection behavior, and the adopted lateral restraint system. Nevertheless, the experimental results indicate that, under the investigated loading and restraint conditions, the HK-type configuration exhibited better overall deformation resistance than the π-type configuration. For large plate girders requiring hanger rod openings, however, the π-type configuration may still be necessary because of structural and construction requirements. A quantitative comparison of the displacement responses between the HK-type and π-type specimens under different eccentric loadings is presented in Table 5.

The arrangement of the strain gauges is shown in Figure 8. Under eccentric loading, the strain gauges located on the web side closer to the eccentric load are denoted by a-x, while those located on the web side farther from the eccentric load are denoted by A-x.

**Figure 10:**
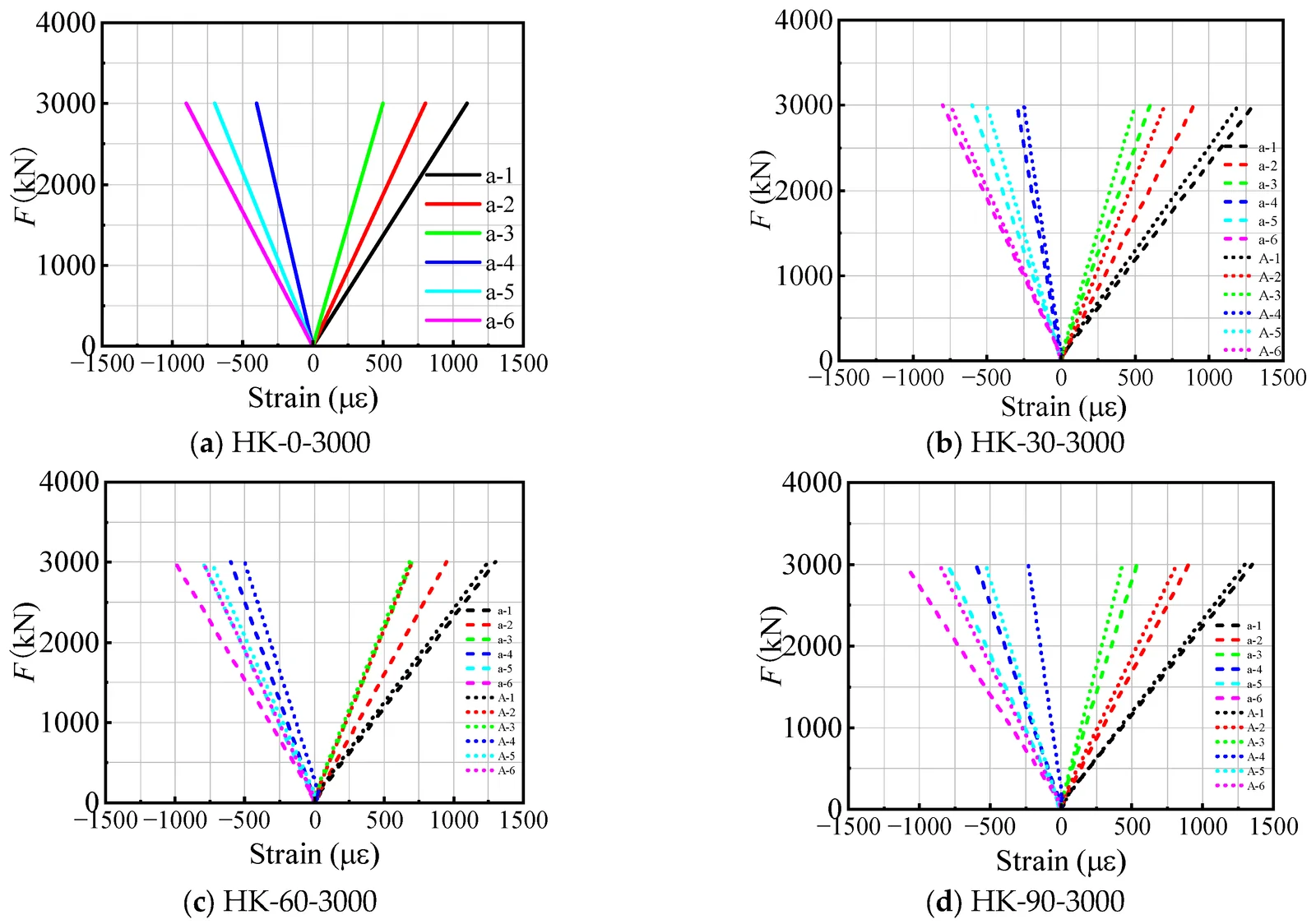

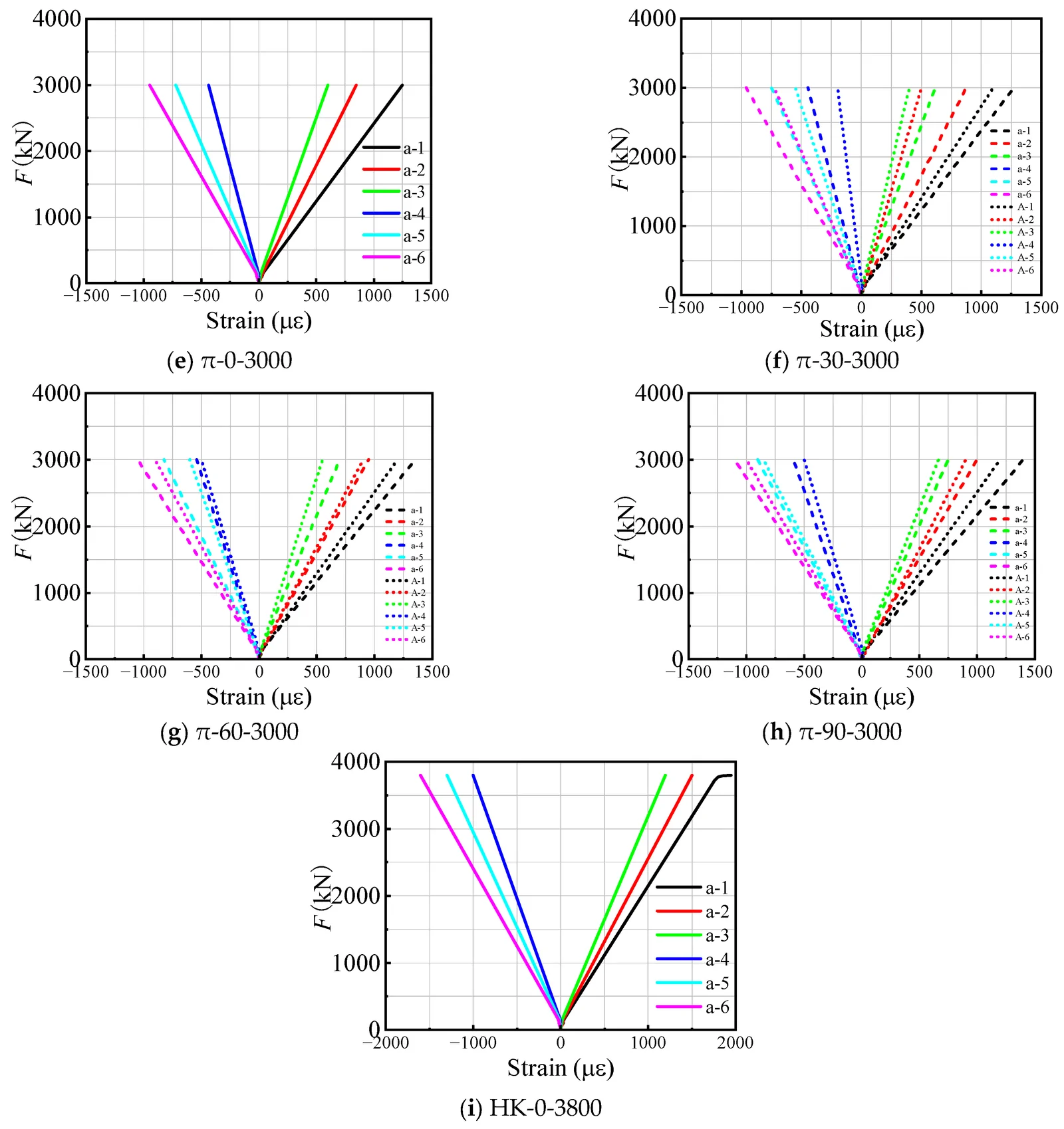
Load–strain curves.

As shown in Figure 10, the web strains of both the HK-type and π-type specimens varied approximately linearly with the applied load within the investigated loading range. Under eccentric loading, differences were observed between the strain responses of the two webs for both types of specimens, indicating an asymmetric sectional response. In general, the strain magnitude of the web closer to the eccentric load differed from that of the opposite web, and the asymmetry tended to become more pronounced with increasing eccentricity. Similar overall trends were observed for the HK-type and π-type specimens, although the strain magnitudes and the degree of asymmetry varied with the girder configuration, gauge position, and loading condition. These results indicate that the web-strain response under eccentric loading is affected by the combined effects of section geometry, bending and shear response, possible torsional deformation, connection behavior, and the lateral restraint system.

Strain gauges were attached to the transverse braces to measure their axial strains during loading. The corresponding axial stresses were calculated using the linear-elastic relation:

*σ*_b_ = *E*_b_*ε*_b_ (1)

where *σ*_b_ is the axial stress of the brace, *E*_b_ is the elastic modulus of the brace material, taken as 210 GPa in the calculation, and *ε*_b_ is the measured axial strain. The four braces were numbered Braces 1–4 from the outer sides toward the inner mid-span region, as indicated in Figure 7, and the same numbering is used in Table 6. The maximum calculated brace stress was 42.88 MPa, which was well below the yield strength of the brace material, confirming that the braces remained within the elastic range during the tests.

Table 7 presents the percentage increase in transverse-brace stress of the π-type specimen relative to the corresponding HK-type specimen under the same eccentricity. As shown in Table 7, under the same eccentric loading conditions, the tested π-type specimens developed higher transverse-brace stresses than the corresponding HK-type specimens, indicating stronger interaction between the π-type girder and the adopted lateral-restraint system.

To quantitatively evaluate the effect of eccentric loading on the deformation resistance of the two tested structural systems, the secant stiffness at the mid-span was calculated based on the load–displacement curves obtained from the tests. The secant stiffness is defined as the ratio of the applied load to the mid-span displacement at a given load level as shown in Equation (2):

(2) $$K=\frac{F}{\delta }$$

To compare the structural stiffness under different eccentric loadings, a uniform load level of 3000 kN was selected. Table 8 presents the mid-span displacement, secant stiffness, and stiffness ratio of HK-type and π-type specimens. The results show that, under the same eccentric loading conditions, HK-type specimens exhibit smaller mid-span displacements and higher secant stiffness than π-type specimens. As the eccentricity increased, the secant stiffness of both HK-type and π-type specimens gradually decreased. To evaluate the relative stiffness variation within each configuration, the secant stiffness was normalized by the corresponding value under concentric loading (*e* = 0). At an eccentricity of 90 mm, the HK-type and π-type specimens retained 89.9% and 90.8% of their respective initial secant stiffness values, corresponding to reductions of 10.1% and 9.2%, respectively. These results indicate that the relative stiffness reductions of the two configurations over the investigated eccentricity range were comparable. Nevertheless, the HK-type specimens maintained higher absolute secant stiffness than the π-type specimens under all corresponding loading conditions.

### 2.5. Simplified Analytical Calculation

To provide a simplified analytical interpretation of the experimentally observed stiffness variation, the response under eccentric loading was represented by an equivalent elastic flexural–torsional compliance model. Since the specimens loaded to 3000 kN remained predominantly within the elastic range, linear elastic superposition was adopted for this first-order calculation. It should be noted that the transverse braces participated in the lateral force-transfer mechanism during the tests; therefore, the following formulation represents the equivalent global response of the girder–restraint system rather than the free torsional response of an unrestrained girder.

When a vertical load *P* acts with a transverse eccentricity *e*, an additional torque *T* = *Pe* is generated. The elastic strain energy of the simplified system can be expressed as

(3) $$U=\frac{P^{2}}{2K_{0}}+\frac{{\left(\mathrm{Pe}\right)}^{2}}{2K_{\theta }}$$

where *K*_0_ is the vertical secant stiffness under concentric loading and *K*_θ_ is an equivalent rotational stiffness accounting for the combined effects of torsional deformation and the adopted lateral-restraint system. According to Castigliano’s theorem, the corresponding generalized vertical displacement is

(4) $$\delta_{\mathrm{an}}=\frac{\partial U}{\partial P}=\frac{P}{K_{0}}+\frac{Pe^{2}}{K_{\theta }}$$

Therefore, the equivalent vertical stiffness under eccentric loading is

(5) $$K_{\mathrm{an}}(e)=\frac{P}{\delta_{\mathrm{an}}}=\frac{1}{\frac{1}{K_{0}}+\frac{e^{2}}{K_{\theta }}}$$

The effective rotational stiffness was evaluated using the concentric-loading result and the test result at the maximum eccentricity of 90 mm:

(6) $$K_{\theta }=\frac{e_{90}^{2}}{\frac{1}{K_{90}}-\frac{1}{K_{0}}}.$$

Based on the secant stiffness values in Table 8, the resulting effective rotational stiffnesses are 1.38 × 10^7^ kN·mm/rad for the HK-type specimen and 1.25 × 10^7^ kN·mm/rad for the π-type specimen. Using these values, the stiffnesses at eccentricities of 30 and 60 mm were calculated and compared with the experimental results, as summarized in Table 9.

Considering the displacement, secant-stiffness, and transverse-brace responses together, the tested HK-type system exhibited smaller vertical displacements, higher absolute secant stiffness, and lower transverse-brace stresses than the corresponding π-type system under the investigated loading and restraint conditions. These observations indicate better overall deformation resistance of the tested HK-type system within the scope of the present experiments.

From an engineering perspective, where deformation control and reduced demand on the lateral-restraint system are primary considerations under similar eccentric-loading conditions, the tested HK-type configuration may be advantageous. However, where openings are required for hanger rods, piping, erection, or other functional requirements, the π-type configuration may remain necessary.

## 3. Conclusions

In this study, HK-type and π-type large plate girder specimens were tested under concentric and eccentric loading conditions. The deformation behavior, strain distribution, transverse brace response, and secant stiffness were investigated. The main conclusions are as follows:

1. Under a load of 3000 kN, the HK-type specimen remained basically in the elastic stage. When the load increased to 3800 kN, the specimen entered the elastic–plastic stage. During the tests, no obvious lateral instability was observed, indicating that the specimen had good overall stability under the investigated loading conditions.
2. Eccentric loading affected the displacement response of both HK-type and π-type specimens. As the eccentricity increased from 0 mm to 90 mm, the mid-span displacement increased gradually. Under the same eccentric loading conditions, the HK-type specimens exhibited smaller mid-span displacements than the π-type specimens, indicating better deformation resistance.
3. The secant stiffness results show that eccentric loading reduced the vertical deformation resistance of both configurations. At the load level of 3000 kN, the secant stiffness of the HK-type specimen decreased from 192.31 kN/mm to 172.81 kN/mm as the eccentricity increased from 0 mm to 90 mm, while that of the π-type specimen decreased from 156.57 kN/mm to 142.18 kN/mm. The secant stiffness of both configurations decreased with increasing eccentricity. From *e* = 0 to 90 mm, the normalized secant stiffness decreased to 89.9% for the HK-type specimen and 90.8% for the π-type specimen, indicating comparable relative stiffness reductions within the investigated eccentricity range. However, the HK-type specimens consistently exhibited higher absolute secant stiffness than the corresponding π-type specimens.
4. Under eccentric loading, differences developed between the strain responses of the two webs, indicating an asymmetric sectional response. The degree of asymmetry generally increased with loading eccentricity.
5. The transverse-brace stresses increased with loading eccentricity. Under the same eccentric loading conditions, the π-type specimens generally developed higher brace stresses than the corresponding HK-type specimens, indicating stronger interaction between the π-type girders and the adopted lateral restraint system.
6. Overall, under the investigated loading and restraint conditions, the HK-type specimens exhibited smaller vertical displacements, higher absolute secant stiffness, and lower transverse-brace stresses than the corresponding π-type specimens. These results characterize the comparative global response of the two tested girder–restraint systems.

Within the scope of the present tests, the results indicate that the HK-type configuration exhibited smaller vertical displacements, higher absolute secant stiffness, and lower transverse-brace stresses than the corresponding π-type configuration. These observations are limited to the tested model geometry, eccentricities of 0–90 mm, the adopted four-brace lateral-restraint system, and the investigated load range. The specimens tested to 3000 kN were used primarily to characterize comparative response within the predominantly elastic range rather than to determine ultimate capacity. Therefore, the results may be used as comparative experimental evidence when evaluating HK-type and π-type configurations under similar loading and restraint conditions, but they should not be directly extrapolated to unrestrained girders or full-scale approximately 30 m boiler girders without additional validation of similitude, restraint characteristics, connection behavior, and scale effects.

## Figures and Tables

**Table 1:** Summary of test specimens and final applied loads.

Specimens	Eccentricity (mm)	Final Load (kN)
HK-0-3000	0	3000
HK-30-3000	30	3000
HK-60-3000	60	3000
HK-90-3000	90	3000
HK-0-3800	0	3800
π-0-3000	0	3000
π-30-3000	30	3000
π-60-3000	60	3000
π-90-3000	90	3000

**Table 2:** Basic sectional properties and nominal steel quantities of the HK-type and π-type configurations.

Property	HK-Type	π-Type	π/HK
Overall depth *h* (mm)	1760	1760	1.000
Overall width *b* (mm)	400	400	1.000
Gross steel area *A* (mm^2^)	45,184	41,344	0.915
Centroid from bottom *y*_c_ (mm)	880.0	960.8	-
Major-axis second moment *I*_x_ (mm^4^)	1.890 × 10^10^	1.572 × 10^10^	0.832
Minor-axis second moment *I*_y_ (mm^4^)	5.059 × 10^8^	4.941 × 10^8^	0.977
Nominal steel mass per unit length (kg/m)	354.7	324.6	0.915

**Table 3:** Summary of material properties of steel plates.

*t* (mm)	Parameter	Specimen 1	Specimen 2	Specimen 3	Mean	SD	CV (%)
4	*E*	200.42	201.95	203.12	201.83	1.35	0.67
4	*f*_y_	409.80	421.15	429.74	420.23	10.00	2.38
4	*f*_u_	610.70	625.20	634.48	623.46	11.99	1.92
6	*E*	200.63	202.08	203.80	202.17	1.59	0.78
6	*f*_y_	358.10	369.85	383.31	370.42	12.61	3.41
6	*f*_u_	658.30	675.80	692.79	675.63	17.25	2.55
8	*E*	202.40	203.52	204.94	203.62	1.27	0.63
8	*f*_y_	391.60	400.95	411.47	401.34	9.94	2.48
8	*f*_u_	598.20	611.10	621.75	610.35	11.79	1.93
15	*E*	197.80	199.25	201.03	199.36	1.62	0.81
15	*f*_y_	375.20	387.70	399.15	387.35	11.98	3.09
15	*f*_u_	617.80	632.40	644.18	631.46	13.22	2.09
20	*E*	198.85	200.18	201.75	200.26	1.45	0.72
20	*f*_y_	399.00	410.80	421.22	410.34	11.12	2.71
20	*f*_u_	621.00	634.80	647.79	634.53	13.40	2.11
28	*E*	199.80	201.65	203.77	201.74	1.99	0.98
28	*f*_y_	515.20	529.50	546.68	530.46	15.76	2.97
28	*f*_u_	666.00	692.00	713.59	690.53	23.83	3.45

**Table 4:** Measured mid-span displacement under different loading conditions.

Specimens	Mid-Span Displacement (mm)	Displacement Near the Concentrated Load (mm)	Displacement Away from the Concentrated Load (mm)
HK-0-3000	15.60	15.61	15.60
HK-30-3000	16.28	16.61	16.07
HK-60-3000	16.73	17.30	16.28
HK-90-3000	17.36	17.66	17.15
HK-0-3800	22.31	22.30	22.33
π-0-3000	19.17	19.2	19.12
π-30-3000	19.53	19.72	19.32
π-60-3000	19.69	20.03	19.56
π-90-3000	21.10	21.69	20.39

**Table 5:** Comparison of displacement responses between HK-type and π-type specimens under different eccentric loadings.

Specimens	Mid-Span Displacement Increase (%)	Near-Side Displacement Increase (%)	Far-Side Displacement Increase (%)
π-0-3000	22.88	23.00	22.56
π-30-3000	19.96	18.72	20.22
π-60-3000	17.69	15.78	20.15
π-90-3000	21.54	22.82	18.89

**Table 6:** Measured stresses in transverse braces under different loading conditions.

Specimens	Brace 1 (MPa)	Brace 2 (MPa)	Brace 3 (MPa)	Brace 4 (MPa)
HK-0-3000	1.02	0.60	1.00	0.37
HK-30-3000	10.3	6.28	6.97	10.77
HK-60-3000	12.87	7.52	8.43	13.25
HK-90-3000	15.25	9.91	10.85	16.57
HK-0-3800	1.09	0.27	0.34	0.87
π-0-3000	1.42	1.33	0.60	1.58
π-30-3000	28.05	25.34	27.42	23.62
π-60-3000	32	33.95	35.48	27.82
π-90-3000	42.88	37.62	40.42	40.24

**Table 7:** Comparison of measured stresses in transverse braces between HK-type and π-type specimens under different eccentric loadings.

Specimens	Brace 1 Stress Increase (%)	Brace 2 Stress Increase (%)	Brace 3 Stress Increase (%)	Brace 4 Stress Increase (%)
π-30-3000	172.33	303.50	293.40	119.31
π-60-3000	148.64	351.46	320.88	109.96
π-90-3000	181.18	279.62	272.53	142.85

**Table 8:** Secant stiffness under different loading conditions.

Loading Condition	Mid-Span Displacement (mm)	Secant Stiffness (kN/mm)	Normalized Secant Stiffness (%)
HK-0-3000	15.6	192.31	100%
HK-30-3000	16.28	184.28	95.8%
HK-60-3000	16.73	179.32	93.2%
HK-90-3000	17.36	172.81	89.9%
π-0-3000	19.17	156.57	100%
π-30-3000	19.53	153.66	98.1%
π-60-3000	19.69	152.34	97.3%
π-90-3000	21.10	142.18	90.8%

**Table 9:** Comparison between experimental and simplified analytical stiffnesses.

Specimen	*e* (mm)	Experimental *K* (kN/mm)	Analytical *K* (kN/mm)	Difference (%)
HK-type	30	184.28	189.93	3.1
HK-type	60	179.32	183.13	2.1
π-type	30	153.66	154.83	0.8
π-type	60	152.34	149.83	1.6

## Data Availability

The original contributions presented in this study are included in the article. Further inquiries can be directed to the corresponding author.
